# 

**Published:** 1835-04-01

**Authors:** 


### CONTENTS

OF THE

### MEDICO-CHIRURGICAL REVIEW,

No. XLIV. APRIL 1, 1835.

[BEING No. IV. OF A DECENNIAL SERIES.]

### REVIEWS.

I.

Treatise on the Formation, Constituents, and Extraction of the Urinary Calculus, &c. &c. By J. G. Crosse, Esq. .. .. .. .. .. .. .. .. .. .. .. 305

II.

Medico-Chirurgical Transactions, Vol. XVIII.

I. On Irritation of the Spinal Cord and its Nerves, in connexion with Disease of the Kidneys. By Ed. Stanley, Esq. .. .. .. .. .. .. .. .. .. .. 353

II. On Malignant Tumors connected with the Heart and Lungs. By John Sims, M.D. .. .. .. .. .. .. .. .. .. .. .. .. .. .. .. .. 357

III.

Sciential Medicine—a New System of Philosophy. By Thomas Eden, M. R. C. S. .. 362

IV.

Pathology of the Uterus and its Appendages. By Robert Lee, M.D. .. .. .. .. 364

V.

An Inquiry into the Nature and Properties of the Blood in Health and Disease. By the late C. T. Thackrah—(New Edition) .. .. .. .. .. .. .. .. .. .. 368

VI.

Anatomical Description of the Parts concerned in Inguinal and Femoral Hernia. Translated from the French of M. Jules Cloquet .. .. .. .. .. .. .. .. 382

VII.

Clinique Medicale, &c. By M. Andral, of La Charité .. .. .. .. .. .. .. .. 398

VIII.

The Philosophy of Health; or, an Exposition of the Physical and Mental Constitution of Man, &c. By Southwood Smith, M.D. .. .. .. .. .. .. .. .. .. 421

IX.

On Dysentery, its Forms and Consequences. By James Annesley, Esq. .. .. .. 425

X.

Transactions of the Medico-Chirurgical Society of Edinburgh .. .. .. .. .. .. 429

I. Hemicrania .. .. .. .. .. .. .. .. .. .. .. .. .. .. .. 430

II. Inflammation of the Orbital Lining .. .. .. .. .. .. .. .. .. .. 430

III. Cases of Hydrocephalus .. .. .. .. .. .. .. .. .. .. .. .. .. 430

IV. Formation of Hydatids .. .. .. .. .. .. .. .. .. .. .. .. .. 431

V. Rupture of Biceps Flexor Cubiti .. .. .. .. .. .. .. .. .. .. .. 432

VI. Influenza in Edinburgh .. .. .. .. .. .. .. .. .. .. .. .. 432
VII. Epidemic Scarlatina in Edinburgh .. .. .. .. .. .. .. .. .. .. 433

XI.

On the Preparation and Medicinal Employment of Aconitine. By Dr. Turnbull .. 434

XII.

Outlines of Botany. By Professor Burnett, F.L.S. .. .. .. .. .. .. .. .. 436

XIII.

A Treatise on Insanity, and other Disorders of the Mind. By J. C. Prichard, M.D... 442

### PERISCOPE.

PART I.

### Spirit of the English Periodicals, and Notices of English Medical Literature.

1. Extraordinary Case of Tetano-Epileptic Convulsions, with the Post-mortem Examination, &c. By James Johnson, M.D. .. .. .. .. .. .. .. .. .. .. 449
2. Acute Nephritis. By M. Le Moine .. .. .. .. .. .. .. .. .. .. .. 452
3. Pathology of the Urine. By Dr. Bostock .. .. .. .. .. .. .. .. .. 454
4. A peculiar Affection of the Lungs of Infants. By M. Joerg .. .. .. .. .. 460
5. On the Connexion between Acute and Chronic Rheumatism. By Dr. J. Johnson 460
6. Remarkable Case of Diseased Heart. By Dr. Seymour .. .. .. .. .. .. 461
7. Poisoning by Arsenic .. .. .. .. .. .. .. .. .. .. .. .. .. .. 463
8. On the Signs of Pregnancy. By Dr. Ingleby .. .. .. .. .. .. .. .. .. 468
9. The Gums, their Diseases and Sympathies. By Mr. Waite .. .. .. .. .. 474
10. Irrenaulstadt—a German Lunatic Asylum on the Rhine .. .. .. .. .. .. 476
11. Medical Problems. By Dr. Griffin.—Remarkable Case of Enteritis .. .. .. 477
12. Dr. Mateer on the injurious Effects of Salt.. .. .. .. .. .. .. .. .. 480
13. Dr. Churchill's Cases of Uterine Inflammation .. .. .. .. .. .. .. .. 481
14. Practical Observations on Cholera. By Mr. Henry George .. .. .. .. .. 483
15. On Renal Diseases. By the Editor .. .. .. .. .. .. .. .. .. .. .. 484
16. Dentology.
1. Mr. Bell on the Teeth.. .. .. .. .. .. .. .. .. .. .. .. 487
2. Mr. Brown's Dentologia .. .. .. .. .. .. .. .. .. .. .. 488
17. Mr. Liston on Diseases of the Rectum .. .. .. .. .. .. .. .. .. .. 489
18. On Endermic Medication. By Dr. Sigmond .. .. .. .. .. .. .. .. 492
19. Dr. M'Cormac on Fever .. .. .. .. .. .. .. .. .. .. .. .. .. 496

PART II.

### Spirit of the Foreign Periodicals.

20. State of Medical Opinion in Paris .. .. .. .. .. .. .. .. .. .. .. 497
21. Purulent Alteration of the Blood .. .. .. .. .. .. .. .. .. .. .. 500
22. Traumatic Phlebitis .. .. .. .. .. .. .. .. .. .. .. .. .. .. 503
23. On the Nature and Treatment of Acute Rheumatism. By Dr. Barthelemy (of Paris) 505
24. On the Acarus Scabiei. By Messrs. Blainville and Dumeril .. .. .. .. .. 507
25. Medical Statistics of Algiers.. .. .. .. .. .. .. .. .. .. .. .. 509
26. On the Treatment of Amenorrhœa by Irritation of the Mamma .. .. .. .. 511
27. On the Treatment of Erectile Tumors by means of Caustic .. .. .. .. .. 512

28. Partial Ossification of Muscles .. .. .. .. .. .. .. .. .. .. .. .. .. 514
29. Poisoning by Oxymur. Hydrarg .. .. .. .. .. .. .. .. .. .. .. .. .. .. 515
30. Some Subjects of Medical Jurisprudence .. .. .. .. .. .. .. .. .. 518
1. Death from Hanging .. .. .. .. .. .. .. .. .. .. .. .. .. .. .. 518
2. Difficulty of ascertainiug the Cause of Death in some Cases .. .. .. .. 519
31. Monomania, inducing to murder.. .. .. .. .. .. .. .. .. .. .. .. .. 520
32. Detection of Poison Twelve Months after Death . .. .. .. .. .. .. .. .. 521
33. Detection of same Seven Years after Death .. .. .. .. .. .. .. .. .. 523
34. Impotence—Affiliation.. .. .. .. .. .. .. .. .. .. .. .. .. .. .. 523
35. On Epidemics .. .. .. .. .. .. .. .. .. .. .. .. .. .. .. .. 524
36. The Chloruret of Lime as a Disinfecting Agent.. .. .. .. .. .. .. .. .. 526
37. Suspected Infanticide (Consultation) .. .. .. .. .. .. .. .. .. .. .. 527
38. Poisoning by Belladonna .. .. .. .. .. .. .. .. .. .. .. .. .. . 528
39. Laceration of the Diaphragm, with several Cases .. .. .. .. .. .. .. .. 529
40. Appeal of the Parisian Surgeons respecting Hospital Appointments.. .. .. .. 533
41. Souvenir du Cholera! a satirical Poem, entitled "Nemesis Médicale!!" .. .. 535
42. On the Responsibility of Medical Men .. .. .. .. .. .. .. .. .. .. .. 536
43. Fees of Medical Men—a Legal Claim in France . .. .. .. .. .. .. .. .. 538
44. On the Desiccation of the Umbilical Cord .. .. .. .. .. .. .. .. .. .. 540
45. Exfoliation of the Epidermis in Infants .. .. .. .. .. .. .. .. .. .. .. 541
46. Memoranda respecting Cataract .. .. .. .. .. .. .. .. .. .. .. .. .. 542
47. Medical Superstitions in some Parts of France .. .. .. .. .. .. .. .. .. 543
48. Rhinoplastic Operation, by Dieffenbach, at La Pitié.. .. .. .. .. .. .. .. 544

### PART III.

### Clinical Review and Hospital Reports.

GLASGOW ROYAL INFIRMARY.

49. Report on Tumors. By Dr. M'Farlane .. .. .. .. .. .. .. .. .. .. .. 545
1. Tumors of the Head and Neck, with numerous Cases .. .. .. .. .. .. 546
2. On Tumors of the Mamma, with Cases and Comments .. .. .. .. .. .. 551

ST. GEORGE'S HOSPITAL.

50. Remarkable Distortion of the Cervical Vertebræ, with Clinical Remarks. By Cæsar Hawkins, Esq.. .. .. .. .. .. .. .. .. .. .. .. .. .. .. .. .. 557

LIVERPOOL OPHTHALMIC INFIRMARY.

51. Report of the Practice for the Year 1834. By Mr. Neill . .. .. .. .. .. .. 559
1. Amaurosis .. .. .. .. .. .. .. .. .. .. .. .. .. .. .. .. .. 560
2. Cataract .. .. .. .. .. .. .. .. .. .. .. .. .. .. .. .. .. 562
3. Contusions and Wounds of the Eye .. .. .. .. .. .. .. .. .. .. .. 562
4. Pustules of the Cornea .. .. .. .. .. .. .. .. .. .. .. .. .. .. 563
5. Strumous Corneitis .. .. .. .. .. .. .. .. .. .. .. .. .. .. .. 563
6. Fistula Lachrymalis .. .. .. .. .. .. .. .. .. .. .. .. .. .. .. 564
52. Sir B. Brodie's Lecture on Hæmorrhoids .. .. .. .. .. .. .. .. .. .. .. 564
Prolapsus of the Rectum.. .. .. .. .. .. .. .. .. .. .. .. .. .. .. 571
Excrescences of the Rectum .. .. .. .. .. .. .. .. .. .. .. .. .. .. 573
53. Remarks on Ulceration of the Rectum, occurring in connexion with Venereal Secondary Symptoms. By H. J. Johnson, Esq. .. .. .. .. .. .. .. .. .. .. 574
1. Ulceration of the Rectum in connexion with Condyloma .. .. . .. 575
2. Phagedænic Ulceration of the Rectum .. .. .. .. .. .. .. .. .. .. 576

3. Ulceration of the Rectum, with Ulceration of other Mucous Membranes .. 578
4. Ulcers of the Rectum accompanying various Secondary Symptoms .. .. 579

### Miscellanies.

54. Birmingham School of Medicine.. .. .. 581
55. Population Returns .. .. 582
56. Dr. Graves' Introductory Lecture to a Course of Physiology and Pathology.. .. 583
57. Signs of the Times—Death, the Great Leveller .. .. 585
58. Why are we Right-handed? .. .. 587
59. Anatomy Bill.. .. .. 588
60. Hospital for Stone.. .. .. 589
61. Bibliographical Record.. .. .. 591

### Extra-Limites.

62. Messrs. French and Dodd on Cholera.. .. .. 593
63. Horne's Acetum Opii Sedativus .. .. .. 594
64. Advantages of Medical Societies .. .. .. 595
65. New School of Anatomy .. .. .. 596

